# Clinical efficacy of combination therapy of an immune checkpoint inhibitor with taxane plus platinum versus an immune checkpoint inhibitor with fluorouracil plus platinum in the first-line treatment of patients with locally advanced, metastatic, or recurrent esophageal squamous cell carcinoma

**DOI:** 10.3389/fonc.2022.1015302

**Published:** 2022-12-20

**Authors:** Ying Li, Yanyan Ji, Lin Shen, Xudong Yin, Tianyu Huang, Bin Deng, Hong Guo, Yunjiang Wu, Yong Chen

**Affiliations:** ^1^ Department of Radio-Chemotherapy, Affiliated Hospital of Yangzhou University, Yangzhou University, Yangzhou, Jiangsu, China; ^2^ Department of Gastroenterology, Affiliated Hospital of Yangzhou University, Yangzhou University, Yangzhou, Jiangsu, China; ^3^ Department of Thoracic Surgery, Affiliated Hospital of Yangzhou University, Yangzhou University, Yangzhou, Jiangsu, China

**Keywords:** esophageal squamous cell cancer, first-line therapy, immune checkpoint inhibitors, immunotherapy, chemotherapy

## Abstract

**Background:**

Chemotherapy combined with immune checkpoints inhibitors (ICIs) has been established as a standard treatment for locally advanced, metastatic, or recurrent esophageal squamous cell cancer (ESCC). However, the optimal chemotherapy regimen in combination therapy is still unclear.

**Purpose:**

To investigate the efficacy and adverse events of the fluorouracil plus platinum (FP) and taxane plus platinum (TP) regimens in ESCC patients receiving chemo-immunotherapy, we conducted this systematic review and meta-analysis.

**Methods:**

Potentially eligible studies were searched from Medline, Embase, Web of Science, and the Cochrane Library. Pooled rates of overall response rate (ORR), disease control rate (DCR), overall survival (OS), progression-free survival (PFS), and adverse events were compared between ICIs+TP and ICIs+FP groups in ESCC patients receiving first-line chemo-immunotherapy.

**Results:**

A total of 10 clinical trials were included, of which 5 were randomized controlled trials. Compared with chemotherapy alone, chemo-immunotherapy significantly improved the OS of ESCC patients (pooled HR=0.69; 95% CI, 0.63–0.76; p<0.01). Pooled analysis revealed that ESCC patients receiving ICIs+TP had significantly higher ORR, DCR, PFS, and OS rates than those receiving ICIs+FP. No statistically significant difference in the pooled incidence rate of treatment-related death was found (2.3% vs 0.9%, P=0.08). ICIs+TP had significantly higher rates of hematologic toxicity but lower rates of gastrointestinal toxicity than ICIs+FP.

**Conclusions:**

Based on the current data, the first-line treatment using ICIs+TP may be a better option than ICIs+FP in advanced, metastatic, or recurrent ESCC.

## Introduction

Esophageal cancer (EC) is one of the most common digestive system cancers with increasing incidence worldwide. According to GLOBOCAN, EC ranked seventh in terms of incidence and sixth in mortality worldwide in 2020 (1). Over half of the global new cases and deaths of EC occur in China every year, and esophageal squamous cell cancer (ESCC) accounts for approximately 90% of all cases (2). At present, a majority of EC patients are diagnosed at a locally advanced or metastatic stage when curative surgery is unavailable (2). The prognosis of EC remains far from satisfactory with an overall 5-year survival rate of about 20% (3).

Over the past decades, fluorouracil plus platinum (FP) was the standard first-line therapy for advanced or metastatic ESCC with the median overall survival (OS) of 5.5-6.7 months (4–6). Several studies with limited sample sizes have indicated that ESCC patients could also benefit from taxane plus platinum (TP) chemotherapy regimen, with median OS from 13.0 to 17.0 months (7–9). Platinum covalently binds to DNA bases, which can inhibit DNA replication and transcription, thus leading to cancer cell death. Fluorouracil mainly affects DNA synthesis by inhibiting thymidine kinase, consequently exerting its cytotoxic properties. The anti-tumor activity of taxanes is associated with the inhibition of tubulin depolymerization, leading to mitotic arrest. Until now, prospective studies have not been conducted comparing the efficacy of TP regimen versus FP regimen as first-line treatment in advanced or metastatic ESCC.

Recently, immune checkpoint inhibitors (ICIs) based treatment strategy has become a new standard both in first-line and second or later-line treatment in metastatic or recurrence disease. Compared with chemotherapy alone, the incorporation of anti-programmed cell death-1 (PD-1) antibodies into chemotherapy has significantly improved the prognosis of ESCC patients. In the KEYNOTE-590 trial (10), results showed that the median OS was 12.6 months for ESCC patients who received pembrolizumab plus FP chemotherapy and 9.8 months in the placebo plus chemotherapy group (P=0.0006). Results from the ESCORT-1ST trial (11) also showed that ESCC patients could significantly benefit from camrelizumab plus TP chemotherapy, with a median OS of 15.3 months in the camrelizumab group and 12.0 months in the chemotherapy group. Until now, there was no strong evidence concerning the different efficacy between TP and FP chemotherapy in the treatment of ESCC patients. Intriguingly, the median OS in anti-PD-1 antibodies plus TP treatment (15.3 months-17.0 months) was numerically better than that in anti-PD-1 antibodies plus FP treatment (12.6 months-13.2 months) from recent phase III randomized controlled trials (RCTs) (10–14), suggesting that different chemotherapeutic drug combination with ICIs might have different therapeutic efficacy. To date, there have been no RCTs that directly compare the therapeutic efficacy of different chemotherapy regimens in advanced or metastatic ESCC patients who received first-line chemo-immunotherapy. According to the principle of PICOS (participants, interventions, comparisons, outcomes, and study design), we conducted this systematic review and meta-analysis to explore the optimal chemotherapy regimen in the first-line chemo-immunotherapy for locally advanced, metastatic, or recurrent ESCC patients.

## Methods

### Literature search strategy

We searched Medline, Embase, Web of Science, and the Cochrane Library for relevant articles published in English with the deadline of February 8, 2022. All searches were conducted in a combination of theme words and free words, and the topics of the studies were divided into 3 aspects: esophageal cancer, chemotherapy, and immunotherapy. The detailed search terms and strategies are shown in **Supplementary Table 1**.

### Inclusion and exclusion criteria

The inclusion criteria were as follows: 1. Patients had histologically or cytologically confirmed ESCC, and with inoperable, locally advanced, recurrent disease, or distant metastatic disease; 2. Chemo-immunotherapy as first-line therapy for eligible patients; 3. Retrospective or prospective Phase 2 or 3 studies were acceptable. The exclusion criteria were as follows: 1. Studies included patients with adenocarcinomas of the esophageal or gastroesophageal junction, and the outcomes of ESCC patients could not be extracted individually; 2. Chemo-immunotherapy was offered as second-line or later-line treatment; 3. Chemo-immunotherapy was combined with radiotherapy or anti-angiogenic therapy. The lists of references of the included articles were manually checked to add additional possible relevant studies. Only the most complete and up-to-date data was extracted if there were multiple publications.

### Data extraction

Data extraction was conducted by two investigators independently. The primary endpoints were progression-free survival (PFS) and OS, followed by overall response rate (ORR), disease control rate (DCR), and adverse events. Disagreements were resolved by consensus. Kaplan-Meier curves were digitized by busing Engauge Digitizer (version 4.1). Tumor-cell PD-L1 expression ≥1% or combined positive score (CPS) ≥10 was defined as high PD-L1 expression.

### Quality assessment

Included RCTs were assessed for the risk of bias using a modified Jadad scale (15). Methodological index for non-randomized studies (MINORS) (16) was used to evaluate the quality of prospective non-randomized studies.

### Statistical analysis

Forest plots for pooled analysis were drawn using R Studio (version 1.1.463). The pooled HRs for OS and PFS were obtained using the RevMan 5.3 analysis software. Before analysis, all percentage data were Freeman-Tukey Double arcsine transformed. Statistical heterogeneity was quantified using I-square (I^2^) tests, with values >50% indicating heterogeneous. Publication bias was visualized by funnel plots. The Pearson chi-square (χ^2^) test was used to determine statistically significant differences between different groups. P<0.05 was considered statistically different.

## Results

### Literature search and description of the studies

**Figure 1:**
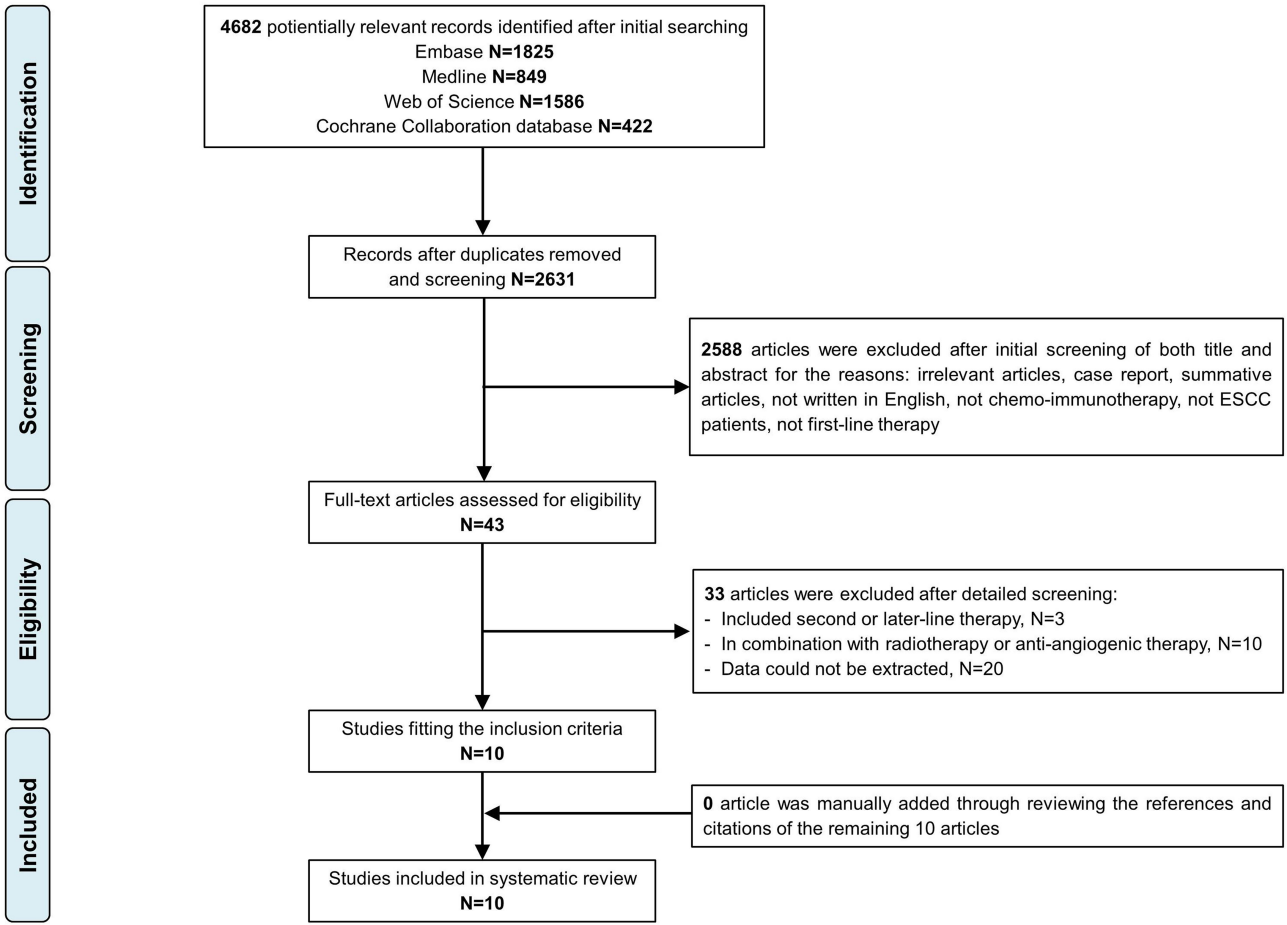
PRISMA flow chart.

**Figure 1** displays the Preferred Reporting Items for Systematic Reviews and Meta-Analyses (PRISMA) study selection flow chart. In total, 4682 citations were identified from Medline, Embase, Web of Science, and the Cochrane Library. A total of 10 articles (1688 ESCC patients) were included in the final analysis.

Details of included studies are summarized in **Table 1**. Three studies were published in abstract form, and the remaining 7 were full-text articles. Most patients (97.8%) received anti-PD-1 therapy. According to different chemotherapy regimens, patients were divided into ICIs+FP and ICIs+TP groups for subgroup analysis. Three studies used both TP and FP regimens. In the ORIENT-15 trial (13), 94% (307/327) patients received the TP regimen, so we classified this study as the ICIs+TP group for the initial analysis, subgroup analysis, and sensitivity analysis further performed. Finally, 647 patients from 4 studies were included in the ICIs+FP group and 980 patients from 5 studies were included in the ICIs+TP group.

**Table 1:** Characteristics of included studies.

Study	Year	Patients	Immunotherapy drug	Chemotherapy regimen	ORR	DCR	mPFS (months)	mOS (months)
Shen et al.	2020	37	CS1001	Cisplatin+5-fluorouracil	67.6%	89.2%	9.0	NR
Xu et al.	2020	15	tislelizumab	Cisplatin+fluorouracil	46.7%	80.0%	10.4	NR
Ren et al.	2020	12	toripalimab	Paclitaxel+Cisplatin	66.7%	91.7%	NR	NR
Wang et al.	2022	257	toripalimab	Paclitaxel+Cisplatin	69.3%	89.1%	5.7	17.0
Sun et al.	2021	274	pembrolizumab	Cisplatin+5- fluorouracil	NA	NA	6.3	12.6
Luo et al.	2021	298	camrelizumab	Paclitaxel+Cisplatin	72.1%	91.3%	6.9	15.3
Lu et al.	2022	327	sintilimab	Cisplatin+5-fluorouracil/Paclitaxel+Cisplatin	66.1%	90.2%	7.2	16.7
Li et al.	2021	61	anti-PD-1 antibodies	Cisplatin+5-fluorouracil/ Taxane +Cisplatin	29.5%	70.5%	7.1	13.5
Wang et al.	2021	86	camrelizumab	Paclitaxel+Nedaplatin	62%	98%	NR	NR
Doki et al.	2022	321	Nivolumab	Cisplatin+fluorouracil	47.4%	79.4%	5.8	13.2

ORR, overall response rate; DCR, disease control rate; mPFS, median progression free survival; mOS, median overall survival; NA, not available; NR, not reached.

### Quality assessment

A total of 10 studies met our inclusion criteria. Five studies were RCTs, and 5 were prospective non-randomized studies. Quality of the RCTs was relatively high, with Jadad scores ranging from 5-7. The remaining prospective studies assessed using the MINORS index scored from 12 to 13 points, which were acceptable for the present systematic review. The results quality evaluation for each study is shown in **Table 2**. All funnel plots were symmetric, suggesting the absence of publication bias (**Supplementary Figure 1**).

**Table 2:** Quality assessment of included studies.

Study	Randomization		Concealment of allocation		Double blinding		Withdrawals and dropouts		Total
A. Modified Jadad scale for included RCT studies.									
Sun JM/2021	2		2		2		1		7
Luo HY/2021	2		2		2		1		7
Lu ZH/2022	2		2		2		1		7
Doki Y/2022	2		2		0		1		5
Wang ZX/2022	1		1		2		1		5
B. MINORS index for included non-randomized studies.									
Study	I	II	III	IV	V	VI	VII	VIII	Total
Shen L/2020	2	2	2	2	0	1	2	2	13
Xu J/2020	2	1	2	2	0	2	2	2	13
Ren C/2020	2	2	2	2	0	2	2	0	12
Li XY/2021	2	2	2	2	0	2	2	0	12
Wang X/2021	2	2	2	2	0	2	2	0	12

numbers I-VIII in heading signified: I, a clearly stated aim; II, inclusion of consecutive patients; III, prospective collection of data; IV, endpoints appropriate to the aim of the study; V, unbiased assessment of the study endpoint; VI, follow-up period appropriate to the aim of the study; VII, loss of follow up less than 5%; VIII, prospective calculation of the study size.

### Meta-analysis of chemo-immunotherapy as first-line treatment for ESCC

**Figure 2:**
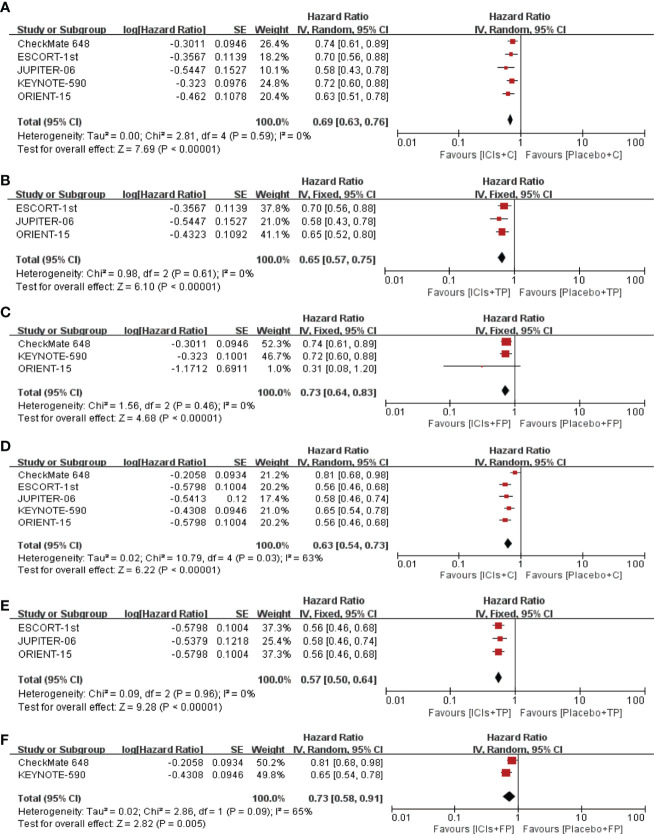
Pooled HR of OS between ICIs+C and placebo+C **(A)**, ICIs+TP and placebo+TP **(B)**, ICIs+FP and placebo+FP **(C)**. Pooled HR of PFS between ICIs+C and placebo+C **(D)**, ICIs+TP and placebo+TP **(E)**, ICIs+FP and placebo+FP **(F)**. HR, Hazard ratio; OS, overall survival; PFS, progression free survival; ICIs, immune checkpoint inhibitors; C, chemotherapy; TP, taxane plus platinum; FP, fluorouracil plus platinum.

A total of 5 RCTs were included in this study. Compared with placebo plus chemotherapy, ICIs plus chemotherapy significantly improved the OS of ESCC patients in whole population (pooled HR=0.69; 95% CI, 0.63–0.76; p<0.01) (**Figure 2**). Same benefits of ICIs plus chemotherapy were also observed in the ICIs+FP group (pooled HR=0.73; 95% CI, 0.64–0.83; p<0.01) and the ICIs+TP group (pooled HR=0.65; 95% CI, 0.57–0.75; p<0.01) (**Figure 2**). Similar results could also be observed in the PFS with pooled HR 0.63 (95% CI, 0.54–0.73; p<0.01) in the whole population, 0.73 (95% CI, 0.58–0.91; p<0.01) in the ICIs+FP group and 0.57 (95% CI, 0.50–0.64; p<0.01) in the ICIs+TP group (**Figure 2**).

### ICIs+TP had significantly better survival than the ICIs+FP

**Figure 3:**
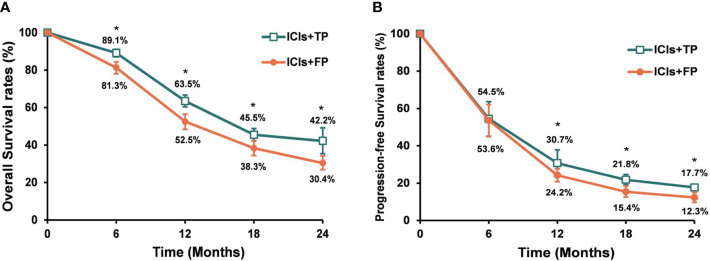
Pooled OS rates **(A)** and PFS rates **(B)** for the ICIs+TP and ICIs+FP groups among ESCC patients who received chemo-immunotherapy. OS, overall survival; PFS, progression free survival; ICIs, immune checkpoint inhibitors; TP, paclitaxel plus cisplatin (TP); FP, 5-fluorouracil plus cisplatin; ESCC, esophageal squamous cell cancer. *ICIs+TP versus ICIs+FP, P<0.05.

The median follow-up time of included studies ranged from 7.1 to 22.6 months. Among the whole population, the pooled median PFS and OS were 6.5 months and 14.9 months, respectively. The pooled 6-, 12-, 18- and 24-month PFS rates were 54.8%, 24.9%, 19.2% and 14.0%, respectively. The pooled 6-, 12-, 18- and 24-month OS rates were 86.5%, 59.2%, 40.3% and 36.2%, respectively (**Supplementary Figure 3**). Subgroup analysis showed that the pooled median PFS and OS were 6.7 months and 16.3 months in the ICIs+TP group, 6.3 months and 12.9 months respectively in the ICIs+FP group. The PFS and OS rates were significantly higher in the ICIs+TP group than in the ICIs+FP group except for 6-month PFS rate (**Figure 3**).

### ICIs+TP had significantly higher treatment response rates than ICIs+FP

Among all ESCC patients who received ICIs+chemotherapy, 1318 patients had measurable lesions. The pooled rates of complete response (CR), partial response (PR), stable disease (SD), and progressive disease (PD) were 2.6%, 54.5%, 29.8%, and 7.5%, respectively. Among the whole population, the pooled ORR and DCR were 61.9% and 91.9% respectively (**Supplementary Figure 2**). Subgroup analysis showed that ORR and DCR were significantly higher in the ICIs+TP group than in the ICIs+FP group (ORR: 71.4% vs 53.3%, P<0.01, respectively; DCR: 94.8% vs 88.0%, P<0.01, respectively) (**Supplementary Figure 4**).

### Analysis of treatment-related adverse events between the ICIs+FP and ICIs+TP

As illustrated in **Table 3**, myelotoxicity and gastrointestinal disorders were the most frequently reported adverse effects. In the whole population, the pooled incidence rates of any grade and ≥3 grade adverse events were 99.0% and 63.9%, respectively. Compared to the ICIs+FP group, the pooled incidence rate of any grade adverse events was significantly higher (99.6% vs 97.3%, P<0.01), but there was no statistical significance between the ICIs+TP and ICIs+FP groups (65.5% vs 66.1%, P=0.84). The pooled incidence rate of treatment-related death was 2.1%, and the difference between the ICIs+TP and ICIs+FP groups was also not found to be statistically significant (2.3% vs 0.9%, P=0.08) (**Table 3**). Subgroup analysis revealed that the incidence rates of anorexia and nausea were significantly lower in the ICIs+TP group than in the ICIs+FP group. However, the incidence rates of leukopenia, neutropenia, thrombocytopenia, and fatigue in the ICIs+TP group were significantly higher than in the ICIs+FP group (**Table 3**). In the ICIs+TP group, the incidence rates of ≥3 grade anorexia and vomiting were significantly lower than in the ICIs+FP group. However, the incidence rates of ≥3 grade anemia, leukopenia, and neutropenia in the ICIs+TP group were significantly higher than in the ICIs+FP group.

**Table 3:** Pooled incidence rates of adverse events in ESCC patients who received chemo-immunotherapy.

Adverse events	Grade	All patients	ICIs+TP	ICIs+FP	P value
Any adverse event		99.0%	99.6%	97.3%	<0.01
Anemia	Any grade	57.1%	59.4%	53.3%	0.05
	Grade ≥ 3	12.1%	13.6%	8.7%	0.02
Leukopenia	Any grade	49.0%	60.9%	29.6%	<0.01
	Grade ≥ 3	14.8%	20.5%	5.3%	<0.01
Neutropenia	Any grade	43.2%	55.6%	20.4%	<0.01
	Grade ≥ 3	24.9%	37.3%	6.9%	<0.01
Thrombocytopenia	Any grade	20.4%	24.1%	10.9%	<0.01
	Grade ≥ 3	1.5%	2.4%	1.4%	0.37
Nausea	Any grade	49.8%	46.1%	59.0%	<0.01
	Grade ≥ 3	0.9%	0.9%	2.2%	0.08
Vomiting	Any grade	27.2%	28.7%	25.2%	0.24
	Grade ≥ 3	2.3%	2.5%	9.7%	<0.01
Diarrhea	Any grade	16.8%	16.7%	18.2%	0.60
	Grade ≥ 3	0.3%	0.8%	0.1%	0.17
Anorexia	Any grade	40.6%	36.5%	55.3%	<0.01
	Grade ≥ 3	0.9%	0.4%	3.1%	<0.01
Fatigue	Any grade	30.8%	32.7%	25.9%	0.02
	Grade ≥ 3	2.5%	3.3%	1.3%	0.07
Treatment-related death		2.1%	2.3%	0.9%	0.08

ESCC, esophageal squamous cell cancer; ICIs, immune checkpoints inhibitors; TP, taxane plus platinum; FP, fluorouracil plus platinum.

The commonly observed immune-related adverse effects were rash (11.4%), pruritus (8.4%), hypothyroidism (7.6%), and pneumonitis (2.0%). Among patients who received camrelizumab (an anti-PD1 antibody), the pooled incidence rate of reactive capillary endothelial proliferation (RCCEP) was 65.5%.

### Prognostic analysis of ESCC patients with high PD-L1 expression

A total of 655 ESCC patients from 4 studies showed PD-L1 high expression. The pooled median PFS and OS were 7.4 months and 15.6 months, respectively. The pooled 6-, 12-, 18- and 24-month PFS were 55.5%, 30.7%, 19.3% and 13.3%, respectively. The pooled 6-, 12-, 18- and 24-month OS were 86.9%, 61.1%, 44.9% and 37.2%, respectively (**Supplementary Figure 5**). Notably, the determination of PD-L1 expression was not the same in different trials. In the CHECKMATE-648 trial, PD-L1 immunohistochemistry (IHC) was performed using the PD-L1 IHC 28-8 pharmDx assay, while PD-L1 expression level was determined using the PD-L1 IHC 22C3 pharmDx assay in the KEYNOTE-590 and the ORIENT-15 trials. In the ESCORT-1ST trial, PD-L1 status was assessed using the 6E8 antibody.

In subgroup analysis, ESCC patients with high PD-L1 expression showed significantly higher 6- and 12-month OS rates in the ICIs+TP group than in the ICIs+FP group. No statistically significant difference was detected in the 18-month OS rate. The pooled median OS was also longer in the ICIs+TP group than in the ICIs+FP group (16.3 months vs 14.7 months) (**Supplementary Figure 6**).

### Sensitivity analysis

**Figure 4:**
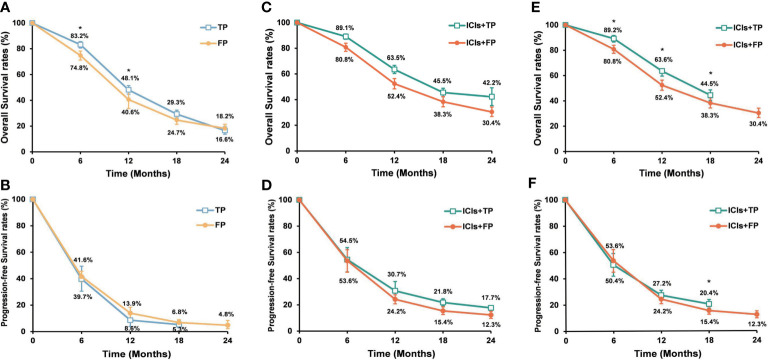
Pooled OS rates **(A)** and PFS rates **(B)** of the TP and FP groups when 5 RCTs were included. Pooled OS rates **(C)** and PFS rates **(D)** of the ICIs+TP and ICIs+FP groups when 5 RCTs were included. Pooled OS rates **(E)** and PFS rates **(F)** of the ICIs+TP and ICIs+FP groups when the ORIENT-15 trial was excluded from the 5 RCTs. OS, overall survival; PFS, progression free survival; ICIs, immune checkpoint inhibitors; TP, taxane plus platinum; FP, fluorouracil plus platinum; RCTs, randomized controlled trial. *TP versus FP or ICIs+TP versus ICIs+FP, P<0.05.

In order to reduce heterogeneity among different studies, treatment efficacy was re-analyzed after excluding studies with small sample sizes or non-RCTs. Therefore, a total of 5 studies (KEYNOTE-590 (10), CHECKMATE-648 (12), ESCORT-1ST (11), JUPITER-06 (14), and ORIENT-15 (13)) were re-analyzed. For ESCC patients who received chemotherapy alone, the pooled analysis showed that patients in the TP group had significantly higher 6- and 12-month OS rates than patients in the FP group. But the 12-month PFS rate was significantly lower in the TP group than in the FP group (**Figure 4**). The pooled median PFS (5.6 months vs 5.7 months) and OS (11.9 months vs 10.3 months) were similar between the TP and FP groups. When ICIs were added to the first-line chemotherapy, pooled analysis showed that PFS and OS rates were significantly higher in the ICIs+TP group than in the ICIs+FP group except for the 6-month PFS rate (**Figure 4**). The pooled median PFS (6.7 months vs 6.0 months) and OS (16.3 months vs 12.9 months) were also longer in the ICIs+TP group as compared with the ICIs+FP group. The pooled ORR and DCR were 71.6% and 93.7% respectively in the ICIs+TP group, which were significantly higher than those reported in the CHECKMATE-648 trial (ORR: 47.4%, DCR: 79.4%). Since a small proportion of patients received ICIs+FP in the ORIENT-15 trial, we further excluded this study to perform the sensitivity analysis again. Our results also showed significantly higher 12-month PFS rate and 6-, 12- and 18-month OS rates in the ICIs+TP group as compared with the ICIs+FP group (**Figure 4**). The difference in pooled ORR (72.1% vs 47.4%, P<0.01) and DCR (91.9% vs 79.4%, P<0.01) remained statistically significant between the ICIs+TP group and those reported in the CHECKMATE-648 trial.

## Discussion

Platinum-based doublet regimens including TP and FP are the most commonly used chemotherapy regimens for the treatment of ESCC. And ICIs combined with either TP or FP has been a new standard strategy based on results from a series of recently published phase III RCTs [KEYNOTE-590 (10), CHECKMATE-648 (12), ESCORT-1ST (11), JUPITER-06 (14), and ORIENT-15 (13)], However, a question concerning which chemotherapeutic regimen could be a better partner with ICIs remains uncertain. In the present study and for the first time, we conducted this meta-analysis and systematic review to explore the difference between ICIs+TP and ICIs+FP in the first-line treatment for ESCC patients. Our results demonstrated higher ORR, DCR, PFS, and OS rates in ESCC patients receiving ICIs+TP than in patients receiving ICIs+FP. These results remained stable throughout the sensitivity analysis. The toxicity profiles were different between the two combinations. Gastrointestinal side effects were common in patients receiving ICIs+FP while hematological toxicities were common in patients receiving ICIs+TP. The incidence rates of treatment-related death were similar between the two groups. These results suggest that both the ICIs+chemotherapy (TP/FP) are effective and the choice must be made looking not only at the treatment efficacy of ICIs but also toxicity and patients’ conditions.

Over the past decades, the FP regimen was the classic combination for the treatment of EC until another effective and broad-spectrum anti-tumor drug, taxane, came to clinical use. Now, platinum plus taxane (paclitaxel or docetaxel) has also shown promising antitumor activity for the treatment of ESCC. A retrospective study showed that the TP regimen had more favorable PFS than the FP regimen but similar OS to the FP regimen in the first-line chemotherapy of advanced ESCC (17). However, this situation might be brought into change by the incorporation of anti-PD-1/PD-L1/CTLA-4 into chemotherapy.

ICIs are believed to have synergistic effects with chemotherapy (18), but how chemotherapy stimulates anticancer immune responses is still not fully understood. Accumulating evidence indicates that chemotherapy could stimulate anticancer immune responses through changes in the surface of tumor cells and modification of the tumor microenvironment (TME), mainly including enhancing the antigenicity or adjuvanticity of cancer cells and modifying the functions or numbers of immune cells (immune effector cells and immunosuppressive cells) (19–22). Because immunogenic cell death (ICD) provides a new opportunity to stimulate tumor specific immune response (22, 23) and thus improve the therapeutic effect of ICIs, chemotherapy drugs (known as one type of ICD inducers) also have the capability of activating ICD-related immunostimulatory pathways such as phosphorylation of eukaryotic translation initiation factor 2 subunit alpha (eIF2α) (24, 25), lysosomal secretion of ATP, translocation of ER chaperones (calreticulin, heat shock protein family A member 1A and heat shock protein 90-α family class A member 1) (22, 26), production of type I IFN through cyclic GMP-AMP synthase/Toll-like receptor 9/TLR3 pathway regulation (27, 28), release of annexin A1 (ANXA1) and high mobility group box 1 (HMGB1) (29). However, different chemotherapy drugs even with similar structures have different ICD-inducing potentials. Tesniere et al. (30) found that oxaliplatin is a more efficient inducer of pre-apoptotic calreticulin exposure than cisplatin in colon cancer cells. Furthermore, Pfirschke et al (31) found that an oxaliplatin-based drug combination (with mafosfamide or cyclophosphamide) could trigger both HMGB1 release and calreticulin exposure (two markers for drug-induced tumor cell immunogenicity), and increase CD8+ T cell: Treg cell ratios inside the tumor from tumor-bearing mice, while other two drug-combinations (paclitaxel-carboplatin and mitoxantrone-mafosfamide) failed to trigger this change in KP lung adenocarcinoma cells (with Kras and Trp53 mutations), suggesting that immunogenic phenotypes can be induced by certain drugs or their combinations in selected cancer. After treated with a chemical library in osteosarcoma U2OS cells, higher levels of p-eIF2a, calreticulin (CALR), and HMGB1 were detected in U2OS cells treated with Paclitaxel/Docetaxel than in U2OS cells treated with Fluorouracil/Capecitabine (24). And Paclitaxel/Docetaxel also has a higher ICD prediction score than Fluorouracil (22, 32). Besides the above effects of chemotherapy drugs on cancer cells, chemotherapy drugs can also stimulate anticancer immunity by directly interacting with a different subset of immune cells. Possible mechanisms include immune system global reset, activation of immune effector cells, and depletion of immunosuppressive cells after exposure to chemotherapy. Hato et al. (33) found that platinum drugs could strongly induce T-cell activation by dendritic cells (DCs), which was associated with the downregulation of PD-L2 on DCs through the IL-4/STAT6 signaling pathway. In EC, Chen et al. (34) found that antitumor immune responses were activated by neoadjuvant paclitaxel plus platinum chemotherapy, thus an immune-activation microenvironment was formed after neoadjuvant chemotherapy (NACT). A higher proportion of immune cells (T and B cells) were detected in patients receiving NACT than in patients not receiving NACT. GSVA analysis showed that NACT could trigger the transition of macrophages from pro-tumor responses in surgery-alone patients to anti-tumor response in NACT patients. However, the enriched pathways of many immune cell subsets between NACT patients and patients with surgery alone were still unknown because of significant heterogeneity and complex regulatory network. Taken together, paclitaxel showed more efficiency in ICD induction and immune-activation microenvironment, which might be one of the possible explanations for the present results that ICIs+TP showed superior synergistic effects over ICIs+FP in the first-line chemoimmunotherapy of ESCC. In addition, the role of immune signatures as potential prognostic and predictive factors has been explored in immune-treated ESCC and other gastrointestinal cancers (35–37). Another important issue to be considered is the difference in immune signatures induced by different chemotherapeutic drugs.

In this study, the most common toxicities were myelosuppression, gastrointestinal disturbances, and immune-related adverse reactions, including rash, pruritus, hypothyroidism, pneumonitis, and RCCEP. It has been reported that 11.7%-45.3% of the ESCC patients who received chemo-immunotherapy discontinued treatment because of adverse effects (11–14). Compared with chemotherapy alone, our pooled analysis showed that the incidence rates of adverse effects were not significantly increased in ESCC patients who received chemo-immunotherapy, suggesting chemo-immunotherapy was a kind of treatment with high efficacy and safety. Subgroup analysis indicated that the ICIs+TP group had significantly higher rates of hematologic toxicity but lower rates of gastrointestinal toxicity than the ICIs+FP group, which was in accordance with the toxicity profile of these drug combinations. Therefore, the toxicity and patients’ conditions should also be considered to balance the benefits and risks for patients before the choice of certain therapeutic regimens. Intriguingly, in Galluzzi et al. review, whole-body physiology influenced by chemotherapy might affect the host-cancer dialogue and therapeutic efficacy (22). Gastrointestinal toxicity caused by chemotherapy not only influences whole-body metabolism but also changes the composition of the gut microbiota, which in turn might be associated with decreased response to chemotherapy as well as ICIs (38, 39). So, another possible explanation for superior synergistic effect of ICIs+TP is partly attributed to the low gastrointestinal toxicity. This hypothesis still needs further study to confirm, and so does the hematologic toxicity.

There are some limitations to the present study. Firstly, only 10 trials were included in this study. Although all funnel plots were symmetric, the Egger or Begg test was not available to formally assess publication bias. Secondly, in this systematic review and meta-analysis, a total of 1651 ESCC patients from 9 studies received anti-PD1 therapy (tislelizumab, toripalimab, pembrolizumab, camrelizumab, sintilimab, and nivolumab) and only 37 patients from 1 study received anti-PD-L1 therapy (CS1001). Although there is no evidence supporting the difference in clinical efficacy between different ICIs, different agents, study designs, detection methods of PD-L1 expression, disease status of study population, and evaluation systems might also be a source of bias. Thirdly, due to the small sample sizes and absence of head-to-head studies comparing ICIs+TP versus ICIs+PF as first-line treatment in inoperable advanced or metastatic ESCC, the results of the present study need more convincing data to confirm. On June 30, 2022, preliminary results of the RATIONALE-306 study (40) have been presented by researchers at World Congress on Gastrointestinal Cancer (available at: https://www.esmo.org/). Similar to our results, tislelizumab plus chemotherapy as first-line treatment of advanced or metastatic ESCC had significantly higher OS (HR=0.66; 95% CI, 0.54–0.80; p<0.01) and PFS (HR=0.62; 95% CI, 0.52–0.75; p<0.01) than chemotherapy alone. The median OS and PFS were 17.2 months and 7.3 months, respectively. However, HRs for OS were similar between tislelizumab plus TP regimen (HR=0.69; 95% CI, 0.54–0.89) and tislelizumab plus FP regimen (HR=0.66; 95% CI, 0.49–0.88). We are looking forward to more detailed subgroup analysis to answer the difference between the two combinations. On May 18, 2022, the phase 3 clinical study ASTRUM-007 (CTR20190911, available at: http://www.chinadrugtrials.org.cn) of serplulimab in combination with FP as a first-line treatment for patients with locally advanced/metastatic ESCC met the co-primary endpoints of PFS and OS in a planned interim analysis. After the data was reported, details on difference in treatment efficacy between ICIs+TP and ICIs+FP in the Chinese population could be obtained. Fourthly, the short duration of follow-up (range: 7.1-22.6 months) may have an impact on survival analysis.

## Conclusion

In conclusion, our analysis showed that chemo-immunotherapy as a first-line treatment could significantly prolong survival when compared with chemotherapy alone in advanced or metastatic ESCC. ICIs+TP could provide a significantly improved response and survival over ICIs+FP. Furthermore, the ICIs+TP group had higher rates of hematologic toxicity but lower rates of gastrointestinal toxicity than the ICIs+FP group. In the future, RCTs directly comparing ICIs+TP versus ICIs+FP are needed, and potential biomarkers require further investigation.

## Data availability statement

The original contributions presented in the study are included in the article/**Supplementary Material**. Further inquiries can be directed to the corresponding author.

## Author contributions

YL and YC designed the study. YL, YJ, and YC searched the literature and screened the relevant studies. YL, YJ, XY, and TH are responsible for data extraction and analyses. YL and BD wrote the manuscript draft. HG, LS, YW, and YC revised the manuscript critically. All authors contributed to the article and approved the submitted version.
