# Supplementary material for: Clinical efficacy of combination therapy of an immune checkpoint inhibitor with taxane plus platinum versus an immune checkpoint inhibitor with fluorouracil plus platinum in the first-line treatment of patients with locally advanced, metastatic, or recurrent esophageal squamous cell carcinoma
Source: Front Oncol. 2022 Dec 20;12:1015302. doi: 10.3389/fonc.2022.1015302 (PMC9808083; doi:10.3389/fonc.2022.1015302)
Supplement: Supplementary file 1 [file DataSheet_1.pdf]

Supplementary Material

1. Supplementary Figures

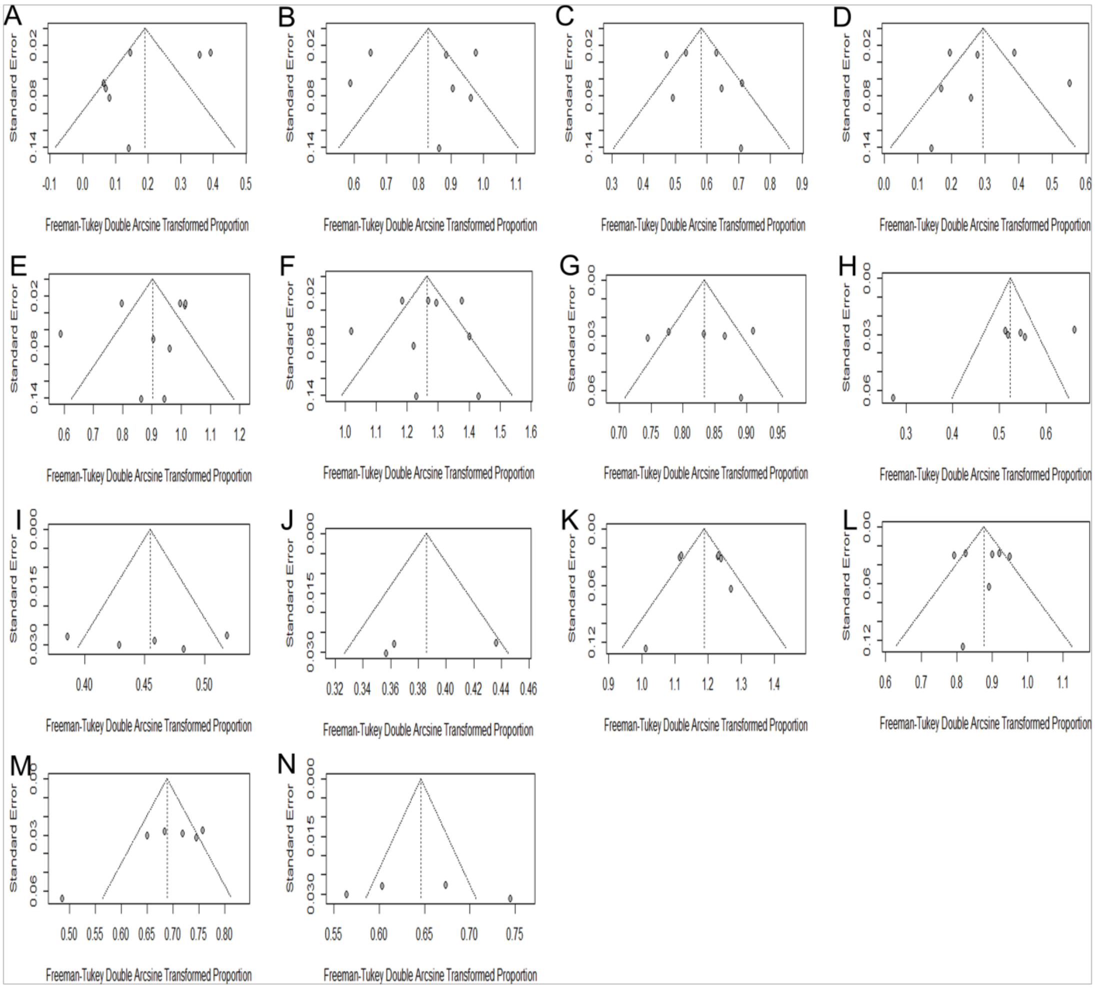

Supplementary Figure 1. Funnel plots for publication bias of CR (A), PR (B), SD (C), PD (D), ORR (E), DCR (F), 1-, 2-, 3-, 5-year PFS rates (G-J), and 1-, 2-, 3-, 5-year OS rates (K-K)

Abbreviations: CR, complete response; PR, partial response; SD, stable disease; PD, progressive disease; ORR, overall response rate; DCR, disease control rate; PFS, progression free survival; OS, overall survival

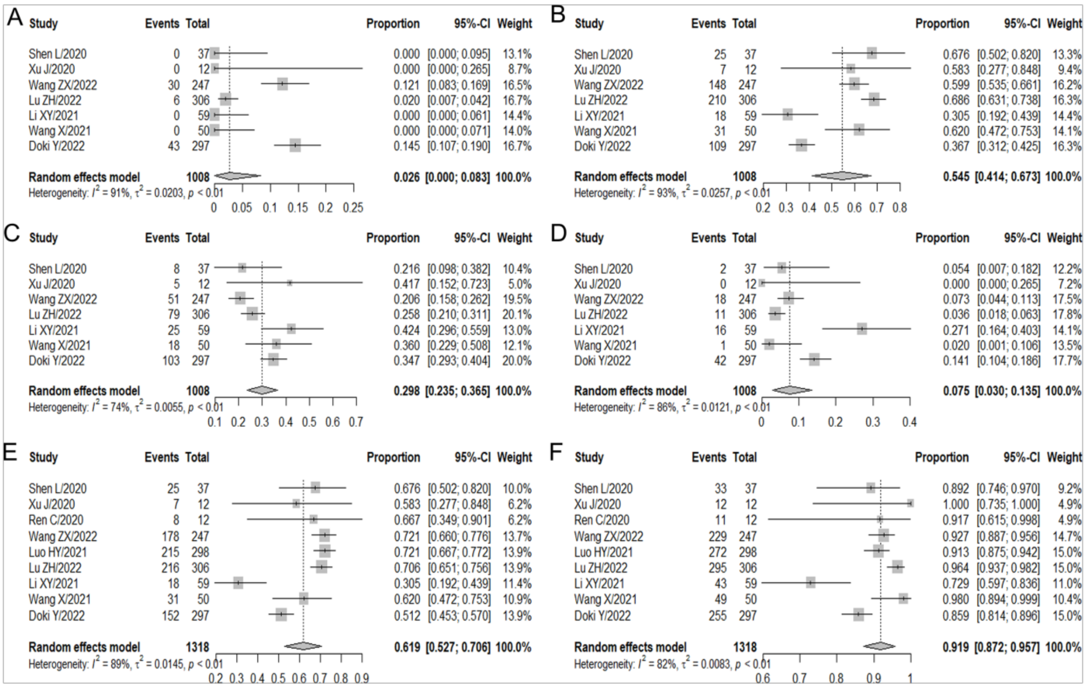

Supplementary Figure 2. Forest plots of CR (A), PR (B), SD (C), PD (D), ORR (E), and DCR (F) in the whole population of ESCC patients who received chemo-immunotherapy

Abbreviations: CR, complete response; PR, partial response; SD, stable disease; PD, progressive disease; ORR, overall response rate; DCR, disease control rate; ESCC, esophageal squamous cell cancer

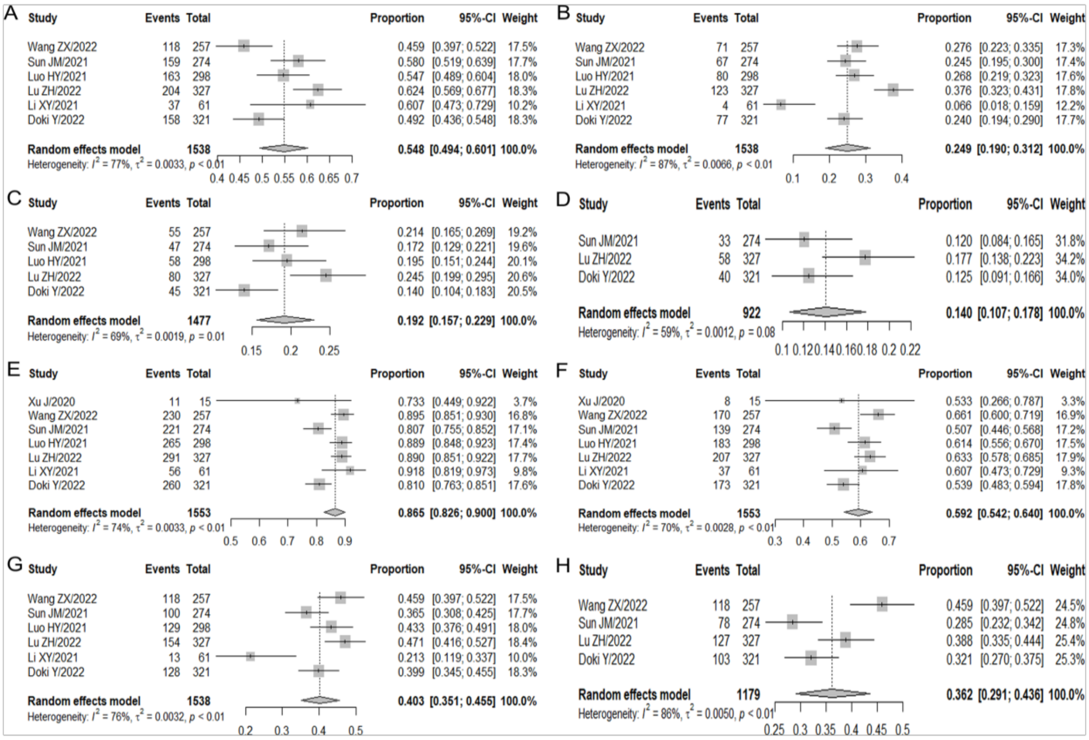

Supplementary Figure 3. Forest plots of 6-, 12-, 18- and 24-month PFS rates (A-D), 6-, 12-, 18- and 24-month OS rates (E-H) in the whole population of ESCC patients who received chemo-immunotherapy

Abbreviations: OS, overall survival; PFS, progression free survival; ESCC, esophageal squamous cell cancer

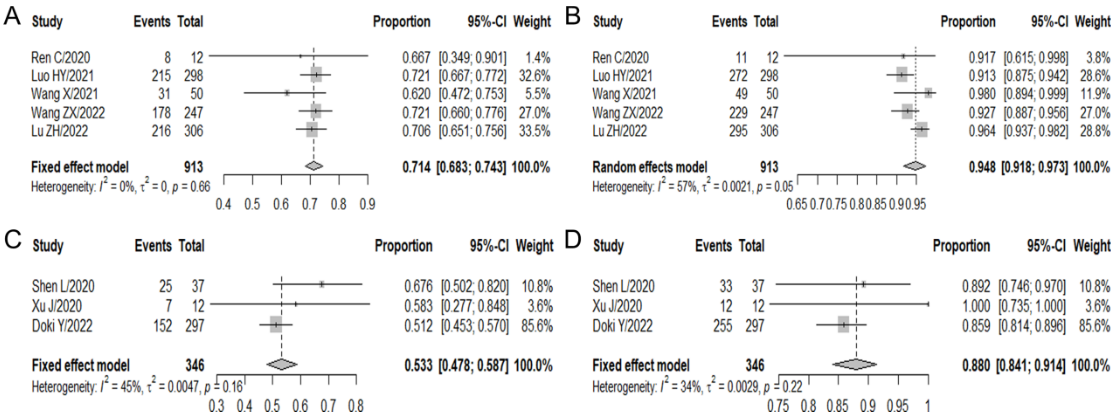

Supplementary Figure 4. Forest plots of ORR (A) and DCR (B) for the ICIs+TP group, ORR (C) and DCR (D) for the ICIs+FP group

Abbreviations: ORR, overall response rate; DCR, disease control rate; ICIs, immune checkpoint inhibitors; TP, paclitaxel plus cisplatin (TP); FP, 5-fluorouracil plus cisplatin

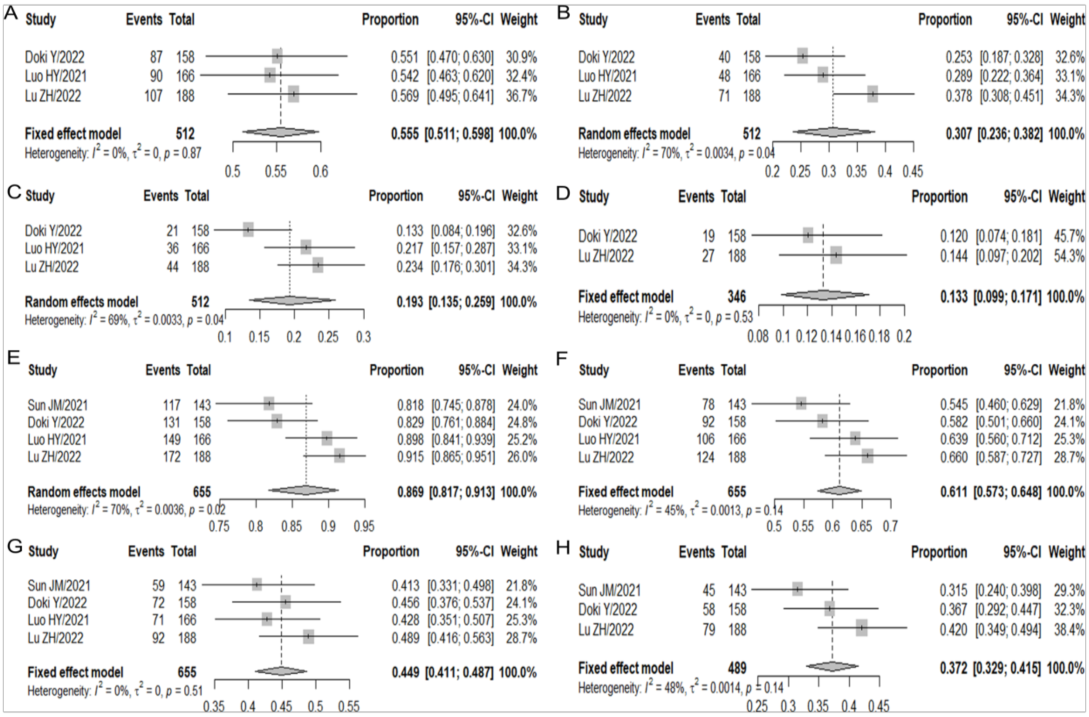

Supplementary Figure 5. Forest plots of 6-, 12-, 18- and 24-month PFS rates (A-D), 6-, 12-, 18- and 24-month OS rates (E-H) in ESCC patients with high PD-L1 expression

Abbreviations: OS, overall survival; PFS, progression free survival; ESCC, esophageal squamous cell cancer

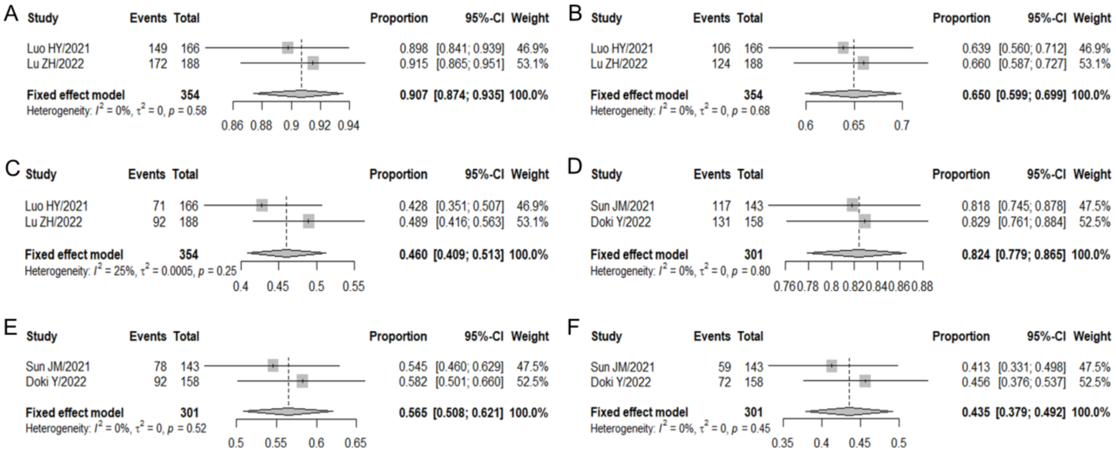

Supplementary Figure 6. Forest plots of 6-, 12-, and 18-month OS rates in the ICIs+TP group (A-C) and ICIs+FP group (D-F) for the treatment of ESCC patients with high PD-L1 expression.

Abbreviations: OS, overall survival; ESCC, esophageal squamous cell cancer; ICIs, immune checkpoint inhibitors; TP, paclitaxel plus cisplatin (TP); FP, 5-fluorouracil plus cisplatin

2. Supplementary Tables

2.1 Supplementary Table 1. Systematic search strategy

1.	((esophageal OR esophagus OR oesophageal OR oesophagus OR gastroesophageal OR oesophagogastric OR esophagogastric) AND (cancer OR cancers OR tumor OR tumour OR tumors OR tumours OR neoplasm OR neoplasms OR malignancy OR malignancies OR adenocarcinoma OR adenocarcinomas OR carcinoma OR carcinomas)) :ti,ab,kw
2.	'esophagus tumor'/exp
3.	(Chemotherapy OR antineoplastic agents) :ti,ab,kw
4.	'Chemotherapy'/exp
5.	(immune checkpoint inhibitor OR ICI OR immune checkpoint blocking agent OR immune checkpoint blockade OR immunotherapy OR immunotherapies OR immunosuppression OR nivolumab OR opdivo OR pembrolizumab OR keytruda OR atezolizumab OR tecentriq OR durvalumab OR imfinzi OR camrelizumab OR sintilimab OR tislelizumab OR toripalimab OR avelumab OR ipilimumab OR tremelimumab OR bavencio OR lambrolizumab OR SHR-1210 OR shr1210 OR JS001 OR IBI308 OR BGBA317 OR BGB-A317 OR Yervoy OR programmed cell death 1 OR PD-1 OR PD1 OR PD 1 OR anti-PD-1 OR programmed cell death-Ligand 1 OR PD-L1 OR PD L1 OR PDL1 OR anti-PD-L1 OR Cytotoxic T lymphocyte antigen-4 OR CTLA-4 OR CTLA4 OR anti-CTLA-4) :ti,ab,kw
6.	'immunotherapy'/exp
7.	(1 OR 2) AND (3 OR 4) AND (5 OR 6)
